# Everybody's Page

**Published:** 1891-04-11

**Authors:** 


### Everybody's Page.

The Parisians have a Pasteur Institute, and last year had 1,546 patients in it and 10 deaths. The Londoners have no Pasteur Institute, but they have a muzzling order, and, it is said, no deaths from hydrophobia. The poor Parisians are feeling a little jealous.

Water by meter sounds a very charming economy, but somehow, from a sanitary point of view, it would seem to have its drawbacks. Is there not even now, with water as it is practically unlimited, a certain portion of the community not altogether lavish about flushing drains? What may we expect when water is *paid for* by meter?

Hysterical people who cannot exist without some new excitement or sensation will probably hie them to Chicago when its exhibition is in full swing. Amongst the novelties, it is stated, there is to be another Eiffel Tower, from the top of which anyone who pleases can be dropped in a car, the velocity of which as it passes through the air will be more than double that of an express train. Those who commit themselves to the tender mercies of the car will be tied down in their seats, and at the end of its descent the car will be plunged into water, and probably the occupants also, who, we hope, will be duly benefitted by such an ingenious but somewhat drastic water cure.

Now that leeches are so rarely used in medical treatment compared with their use a few generations ago, the occupation of the leech-gatherers of Brittany, whence the majority of these bloodthirsty creatures was imported, must have declined wofully. But it is an ill wind that blows no one any good. Those who have once experienced their application and the painful bite resulting, with the gradual distension of the leech until, filled to repletion, it has the grace to retire and digest its meal, are as glad to be quit of them as the household which was so frequently alarmed at the loss of the creature. Yet the visions of a leech crawling on to your legs unaware were needless, for he does not want another meal for a year. What a lot of domestic trouble, household worry, and expense these lucky vampires are saved.

The late Mr. Kavanagh, M.P., was a remarkable instance of the triumph of perseverance over the greatest difficulties. As a writer in *Blackwood's Magazine* states: "The secret of his success in shooting and fishing was, that by constant practice he had trained limbs, which extended only a few inches from the shoulder, to do almost all the work of full-grown arms and hands. Though a square-built man, he could make them meet across his chest, and so tightly that it was impossible to take a sixpence from between them. He had no hooks or other appliances whatever. In shooting he carried a gun without any trigger-guard—a most dangerous practice, it must be owned, though Kavanagh never had an accident. When he wanted to fire, he threw his gun across the left stump, and supported the stock and touched the trigger with his right. It must have taken him a long time to acquire the knack of swinging himself round so as to get in line with a bird or hare going straight from him."

The Chinese are alleged to be fond of mild remedies for diseases, though the names of their mixtures do not sound reassuring. "The liver of the hiding dragon" should certainly prove efficacious in soothing the derangement of the system which occurs during cholera, especially as its component parts are pig's liver mixed with brickdust from the inside of a furnace! A more suitable medicine for an ostrich than for a human being, we should have thought.

How much sanitary science still has to accomplish is only too clearly shown by the computation that fully one quarter of a million deaths occur every year in this country which are preventable. Mr. Wilson Noble considers that there are on the average nearly forty cases of sickness for every death, and that each case of illness lasts on an average eighteen and a half days, which means a loss of seventy-eight and a half million days' labour. This computation does not include extra cases of illness which occur amongst the working classes from exhaustion. If only half these cases are taken into consideration, it will be found that each year the work of a thousand men for one hundred years is lost through illness.

The changes which have come over the method of travelling in this country during the period that Sir Richard Moon was chairman of the North-Western Railway have been more remarkable than the present generation are able readily to conceive. A passenger can now travel from London to Bath in two and a quarter hours for the sum of eight shillings and elevenpence. What a contrast to the time and money expended over the same journey one hundred years ago! Then "flying machines" were advertised to leave Bath at eleven at night, arriving in London the following evening. The fare was twenty-eight shillings, ten pounds weight of luggage only allowed, any amount in excess being charged at the rate of three halfpence a pound. There were also flying wagons, which flew with such dangerous rapidity as to accomplish the journey in three nights and two days!

Between two stools we come to the ground, and the mania for abolishing every sort of religious and moral teaching in Board schools, as opposed to Sectarian teaching, has had a somewhat similar result. "The conduct of life," and the enforcement of the maxim that "each should try to do his duty in the place where he finds himself" are subjects which, as a writer in the *Times* deplores, receive little or no attention. There is a general stampede in these days, each man rushing to attain the greatest benefit for himself, regardless of how he attains it, or the general effect of his individual conduct on society at large. The law of "*meum*" is strongly to the fore, but that of "*tuum*" is not thought equal in importance. So much care has, in fact, been taken that even the simplest morals, the bare outlines of duty and charity, shall not be inculcated into the minds of the present generation in any specific *religious* manner that the outlook for the conduct of future generations is not very promising, unless all the elements which go to make up what may be called "social economy" are to be considered worthy the attention of the present School Board children.

